# Factors associated with the duration of action of dexamethasone intravitreal implants in diabetic macular edema patients

**DOI:** 10.1038/s41598-019-56143-1

**Published:** 2019-12-20

**Authors:** Young Gun Park, Moon Young Choi, Jin-woo Kwon

**Affiliations:** 1 Department of Ophthalmology, Seoul St. Mary’s, Hospital, College of Medicine, Catholic University of Korea, Seoul, South Korea; 2 Department of Ophthalmology, St. Vincent’s, Hospital, College of Medicine, Catholic University of Korea, Seoul, South Korea

**Keywords:** Retinal diseases, Diseases

## Abstract

We designed this study to determine the association between the duration of action of intravitreal dexamethasone implants and aqueous humor biomarkers or optical coherence tomography (OCT) findings of diabetic macular edema (DME) patients. We measured the concentrations of interleukin (IL)-1β, -8, -10, -17; placental growth factor; and vascular endothelial growth factor in the aqueous humor, and identified the number of hyperreflective foci (HF), grades of ellipsoid zone disruptions, and baseline central subfield thicknesses (CSTs) using OCT of patients with DME. The average duration of action of dexamethasone implants was 4.32 ± 1.18 months. In multivariate linear regression analyses, the duration of action was associated with aqueous IL-8 levels and the number of HF (β = −0.016, p = 0.037 and β = −0.073, p = 0.035, respectively). Multivariate logistic regression showed that the number of HF (>10) was significantly associated with a shorter duration (<4 months) of action (odds ratio: 17.17, p = 0.010). The duration of action of intravitreal dexamethasone implants in DME patients was associated with the level of aqueous IL-8 and the number of HF using OCT. Specifically, higher number of HF in the OCT was associated with a shorter duration of action.

## Introduction

Diabetic macular edema (DME) is a common cause of visual disturbance in diabetic retinopathy (DR)^1,2^. It results from breakdown of the blood–retina barrier induced by metabolic changes and inflammation^3–5^.

The grid or focal retinal photocoagulation treatment has been used to treat DME. Laser photocoagulation effectively lowers macular thickness, but can result in permanent visual field defects^6–8^. Vitrectomy has also been performed in DME cases with refractoriness or other pathological conditions such as tractional components^9,10^. However, with studies revealing the essential role of vascular endothelial growth factor (VEGF) in DR, anti-VEGF agents have become the main treatment for DME^11,12^. Intravitreal steroids have also been widely used for several decades^13,14^. Intravitreal triamcinolone acetonide has been used to treat DME, but may lead to increased intraocular pressure, cataract development, and non-infectious endophthalmitis^15^.

Recently, micronized dexamethasone in a biodegradable copolymer has become available. This form of steroid is used to control the inflammation that plays a role in DME pathogenesis. In a previous study, this copolymer resulted in less increase in intraocular pressure compared to triamcinolone, and the increased intraocular pressure was well-controlled with anti-glaucoma eye drops^14^. In terms of efficacy, dexamethasone is more effective at reducing central subfield thickness (CST) and improving visual acuity in DME patients^16^. However, the duration of action differs among patients, so there is no consensus for a follow-up schedule after injection.

Based on these considerations, in the present study, we identified factors associated with the duration of action of dexamethasone intravitreal implants in DME patients, using aqueous humor biomarkers and optical coherence tomography (OCT).

## Results

We enrolled 47 naïve center-involving DME (CIDME) eyes of 47 patients. The mean age was 57.15 ± 7.28 years, and there were 16 males and 31 females. In DR staging, 28 patients had proliferative DR (59.57%) and 19 patients had non-proliferative DR (40.43%). The mean BCVA (best-corrected visual acuity, logMAR) was 0.72 ± 0.25, and the mean CST was 468.02 ± 102.70 µm at baseline. When classifying the DME morphology as cystoid macular edema (CME) or diffuse retinal thickening (DRT), 23 patients were classified as CME and the others were classified as DRT. The systemic and ocular characteristics of the patients enrolled are summarized in Table 1.

**Table 1:** Demographics and clinical characteristics of DME patients.

				N = 47
Systemic factors	Sex (male:female)			16:31
	Age (years)			57.13 ± 7.28
	HbA1C (%)			7.32 ± 0.92
	DM duration (years)			8.00 [3.00;13.50]
OCT findings	Number of HF			9.47 ± 4.79
	Retinal morphology	CME		23 (48.94%)
		DRT		24 (51.06%)
	Presence of SRD			11 (23.40%)
	EZ disruption grade		0	20 (42.55%)
			1	15 (31.91%)
			2	12 (25.53%)
Aqueous humor	IL-1β (pg/mL)			0.98 [0.00;3.49]
	IL-8 (pg/mL)			18.18 [12.71;34.44]
	IL-10 (pg/mL)			0.00 [0.00;0.00]
	IL-17 (pg/mL)			1.80 [0.00;2.56]
	VEGF (pg/mL)			70.44 [33.52;93.59]
	PlGF (pg/mL)			2.14 [0.00;3.79]
Ocular factors	Axial length (mm)			23.29 ± 0.72
	Baseline BCVA (LogMAR)			0.70 [0.50;1.00]
	BCVA after injection (LogMAR)			0.40 [0.30;0.70]
	Baseline CST (µm)			468.02 ± 102.70
	Thinnest CST after injection (µm)			272.77 ± 23.50
	DMR (NPDR:PDR)			19:28

Values are expressed as mean ± SD or median and interquartile range, as appropriate.

DME, diabetic macular edema; HbA1c, glycated hemoglobin; HF, hyperreflective foci; CME, cystoid macular edema, DRT, diffuse retinal thickening; SRD, Serous retinal detachment; EZ, ellipsoid zone; IL, interleukin; VEGF, vascular endothelial growth factor; PlGF, placental growth factor; BCVA, best-corrected visual acuity; CST, central subfield thickness; DMR, DM retinopathy; NPDR, non-proliferative diabetic retinopathy; PDR, proliferative diabetic retinopathy.

**Figure 1:**
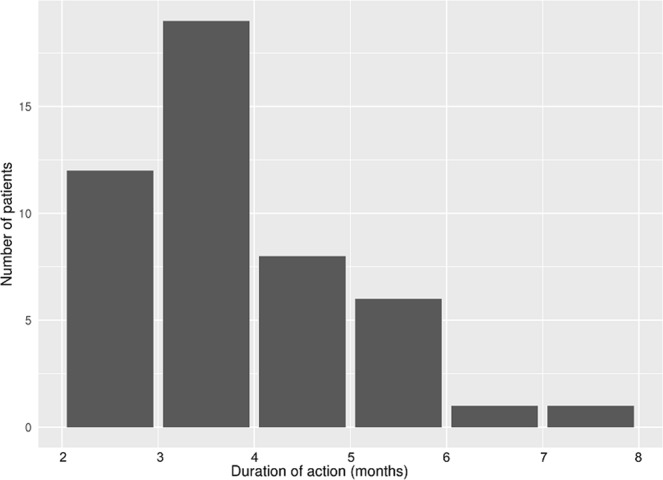
Frequency distribution in the duration of action of dexamethasone intravitreal implants in diabetic macular edema patients.

The average interval between intravitreal dexamethasone implants and recurrence of DME was 4.32 ± 1.18 months. Figure 1 shows the distribution of the interval durations. The average period showed that the lowest CST value was at 2.15 ± 0.66 months after intravitreal dexamethasone implantation. The highest values of intraocular pressure (IOP) occurred at 2.17 ± 0.92 months after implantation, and the average increase was 4.96 ± 2.94 mmHg.

**Figure 2:**
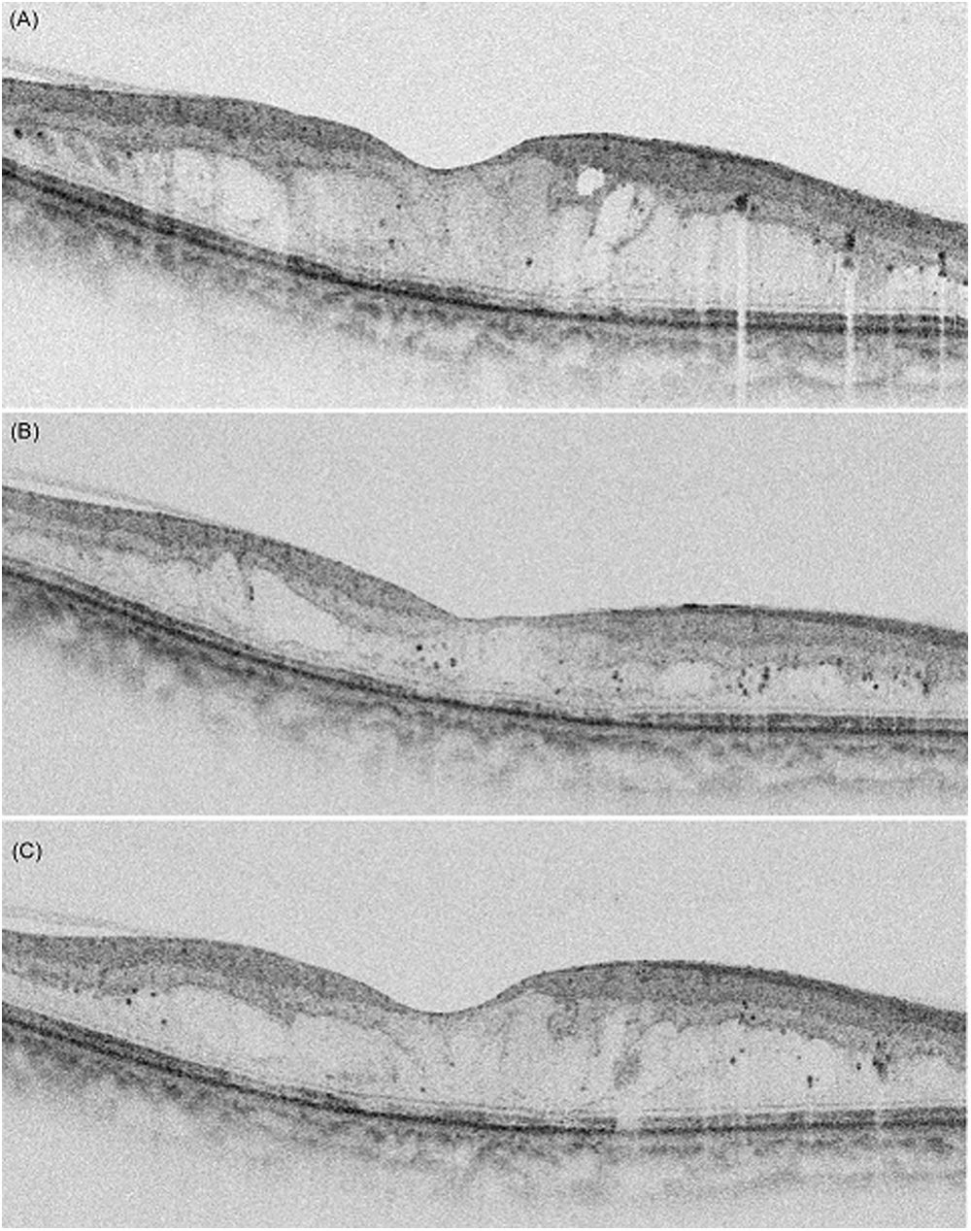
A representative patient who had diabetic macular edema (DME) with multiple hyperreflective foci (HF) and a shorter duration of macular stabilization after dexamethasone implantation. (**A**) The baseline spectral domain-optical coherence tomography (SD-OCT) image shows center-involving DME with multiple HF. (**B**) SD-OCT shows that the DME is decreased at 2 months after intravitreal dexamethasone implantation. (**C**) SD-OCT showing that DME recurred at 3 months after intravitreal dexamethasone implantation.

In the multivariate linear regression analyses for identifying factors related to level of CST reduction after treatments in DME, the aqueous interleukin (IL)-10 level showed significant association (β = 37.31, p = 0.018, Table 2). Factors identified as being associated with the interval are summarized in Table 3. In multivariate linear regression analyses including OCT findings and biomarkers of the aqueous humor, the interval was associated with IL-8 levels of the aqueous humor and the number of hyperreflective foci (HF) using OCT (β = -0.016, p = 0.037 and β = −0.073, p = 0.035, respectively). Multivariate logistic regression for identifying factors affecting a short duration (<4 months) of macular stabilization showed that the number of HF (>10) was significantly associated with a shorter duration of action (odds ratio [OR]: 17.17, p = 0.010, Table 4, Fig. 2).

**Table 2:** Variables associated with the level of CST reduction for diabetic macular edema in linear regression analyses.

		Univariate analyses^*^		multivariate analyses^*†^	
		β ± SE	p-value	β ± SE	p-value
Aqueous humor	IL-1 β level (pg/mL)	3.862 ± 8.566	0.654		
	IL-8 level (pg/mL)	0.194 ± 0.704	0.784		
	IL-10 level (pg/mL)	35.439 ± 16.220	0.034	37.311 ± 15.168	0.018
	IL-17 level (pg/mL)	2.437 ± 8.241	0.769		
	VEGF level (pg/mL)	−0.075 ± 0.226	0.743		
	PlGF level (pg/mL)	−1.423 ± 5.142	0.783		
OCT findings	Number of HF	5.122 ± 3.010	0.096	1.565 ± 3.081	0.614
	EZ disruption grade	42.606 ± 17.087	0.016	19.437 ± 19.978	0.336
	SRD	42.457 ± 34.181	0.221		
BCVA at baseline (logMAR)		144.271 ± 56.287	0.014	115.997 ± 61.766	0.067

^*^Adjusted for age, sex.

CST, central subfield thickness; IL, interleukin; VEGF, vascular endothelial growth factor; PlGF, placental growth factor; HF, hyperreflective foci; EZ, ellipsoid zone; SRD, Serous retinal detachment; BCVA best corrected visual acuity.

^†^R^2^ = 0.277.

**Table 3:** Variables associated with the interval of intravitreal dexamethasone implant and recurrence of diabetic macular edema in linear regression analyses.

		Univariate analyses^*^		multivariate analyses^*†^	
		β ± SE	p-value	β ± SE	p-value
Aqueous humor	IL-1 β level (pg/mL)	−0.091 ± 0.101	0.371		
	IL-8 level (pg/mL)	−0.021 ± 0.007	0.010	−0.016 ± 0.006	0.037
	IL-10 level (pg/mL)	−0.170 ± 0.200	0.401		
	IL-17 level (pg/mL)	0.080 ± 0.097	0.412		
	VEGF level (pg/mL)	−0.001 ± 0.003	0.802		
	PlGF level (pg/mL)	−0.055 ± 0.060	0.369		
OCT findings	Number of HF	−0.111 ± 0.033	0.002	−0.073 ± 0.034	0.035
	EZ disruption grade	−0.113 ± 0.215	0.604		
	CST before injection (μm)	−0.004 ± 0.002	0.017	−0.003 ± 0.002	0.061
	Presence of SRD	0.058 ± 0.411	0.888		

^*^Adjusted for age, sex.

IL, interleukin; VEGF, vascular endothelial growth factor; PlGF, placental growth factor; HF, hyperreflective foci; EZ, ellipsoid zone; CST, central subfield thickness; SRD, Serous retinal detachment.

^†^R^2^ = 0.326.

**Table 4:** Results of logistic regression, effect of a shorter duration of action (<4 months) of intravitreal dexamethasone implantation in DME patients.

		Category	n(%)	Univariate		Multivariate	
				OR (95%CI)	p	OR (95%CI)	p
Sex		Female	31 (65.96%)	Reference			
		Male	16 (34.04)	2.40 (0.61,12.11)	0.238		
Age (years)		≤57	32 (68.09%)	Reference			
		>57	15 (31.91%)	0.99 (0.28, 3.42)	0.989		
HbA1c		≤7	15 (31.91%)	Reference			
		>7	32 (68.09%)	0.69 (0.16, 2.59)	0.598		
DMR stage		NPDR	19 (40.43%)	Reference			
		PDR	28 (59.57%)	0.41 (0.10, 1.49)	0.194		
CST (μm)		≤400	15 (31.91%)	Reference			
		>400	32 (68.09%)	2.62 (0.72, 9.82)	0.143		
EZ disruption		(−)	20 (42.55%)	Reference			
		(+)	27 (57.45%)	1.28 (0.37, 4.45)	0.696		
Number of HF		≤10	27 (57.45%)	Reference		Reference	
		>10	20 (42.55%)	20.46 (3.46, 394.23)	0.006	17.17 (2.80, 344.84)	0.010
Retinal morphology	type	DRT	24 (51.06%)	Reference			
		CME	23 (48.94%)	1.70 (0.50, 6.16)	0.403		
	SRD	(−)	36 (76.60%)	Reference			
		(+)	11 (23.40%)	0.46(0.11, 1.92)	0.277		
IL-1β (pg/mL)		≤0.98	24 (51.06%)	Reference			
		>0.98	23 (48.94%)	0.77 (0.22, 2.65)	0.680		
IL-8 (pg/mL)		≤18.18	24 (51.06%)	Reference		Reference	
		>18.18	23 (48.94%)	4.02 (1.11, 17.15)	0.043	2.82 (0.64, 13.85)	0.177
IL-10 (pg/mL)		<1.60	36 (76.60%)	Reference			
		≥1.60	11 (23.40%)	0.77 (0.19, 3.44)	0.718		
IL-17 (pg/mL)		≤1.80	24 (51.06%)	Reference			
		>1.80	23 (48.94%)	0.52 (0.14, 1.78)	0.302		
VEGF (pg/mL)		≤70.44	24 (51.06%)	Reference			
		>70.44	23 (48.94%)	1.70 (0.50, 6.16)	0.403		
PlGF (pg/mL)		≤2.14	24 (51.06%)	Reference			
		>2.14	23 (48.94%)	1.70 (0.50, 6.16)	0.403		

DME, diabetic macular edema; OR, odds ratio; CI, confidence interval; HbA1c, glycated hemoglobin; EZ, ellipsoid zone; CST, central subfield thickness; HF, hyperreflective foci; CME, cystoid macular edema, DRT, diffuse retinal thickening; SRD, Serous retinal detachment; IL, interleukin; VEGF, vascular endothelial growth factor, PlGF, placental growth factor.

## Discussion

The pathogenesis of DME is complex; ischemia and inflammation are closely associated with each other^4,17^. Several DME treatment options are now available^7,10^; currently, the principal treatment is intravitreal injection of anti-VEGF antibodies or steroids, which are effective and convenient^11,14,16^. Anti-VEGF agents effectively relieve macular edema and have few side effects. Additionally, they could eliminate neovascularization and downgrade DR staging^18,19^. However, steroid implants are more potent and have a longer effect compared to anti-VEGF agents, but they have side effects, including cataract formation and increased IOP^14^. The duration of action of dexamethasone implants differs among patients because they have various systemic and ocular conditions. We suggest that the duration of action of dexamethasone implants could correlate to the degree of activity of DME, in other words, a short period recurrence after treatment could reflect higher activity of DME. Consistent with this possibility, in this study, we first reported that the aqueous IL-8 levels and number of HF were associated with the activity of DME.

IL-8 is a chemokine plays a role in neutrophil chemoattractant and T-cell activator^20^. We had reported that the group who responded poorly to intravitreal anti-VEGF treatments had higher aqueous levels of IL-8, when compared with the group who responded well in another study^21^. In DME, hypoxia causes endothelial and microglial cells to produce IL-8, which is involved in inflammation and neovascularization^22–24^. IL-8 levels are elevated in the aqueous humor of DME patients, which is associated with inflammation involving breakdown of the blood–retina barrier^25,26^. Intravitreal triamcinolone acetonide is effective for patients unresponsive to IVB, and its efficacy is related to IL-8 levels in the aqueous humor^27^. However, one review article suggested that IL-8 may play a role in DME development and may not be adequately controlled by either anti-VEGF antibodies or steroids^28^. In the present study, we showed that the duration of treatment was associated with levels of IL-8 in the aqueous humor. The role played by IL-8 in DME patients, in terms of responsiveness to various treatments, requires further investigations.

HF, detected using OCT as dot shapes, were first described in patients with DME as subclinical features of lipoprotein extravasation that may be precursors to hard exudates^29^. HF have been reported in various retinal diseases, including age-related macular degeneration, retinal vein occlusion, and central serous chorioretinopathy, and are associated with the prognosis of each disease^30–33^. In the case of DME, although some reports have suggested that HF are migrating pieces of retinal pigmented epithelium or degenerated photoreceptor cells^34,35^, recent studies have suggested that they are activated forms of microglia, and may be markers of inflammation^36,37^. Some studies have reported that increased HF could be a poor prognostic factor that results in worse final visual acuity and responsiveness with regard to CST reduction after anti-VEGF treatments in DME patients^38,39^. Our results also suggested that HF may be indicative of DME activity; a higher HF could suggest more recurrence and the need for more treatment.

The IL-8 level and number of HF have something in common with factors that are related with inflammation, especially activated microglial cells^23,24,36^. As many studies reveled microglial cell could be a key cell mediate inflammation in DME^4^, and our study also be one of evidence that prove this hypothesis.

Although IL-10 is representative anti-inflammatory cytokine, it is also associated with pathologic angiogenesis in the eye^40^. Our study showed the aqueous IL-10 level was positively correlated with the level of CST reduction, on the other hand, on the other hand, another study reported that aqueous humor of IL-10 was negatively associated with BCVA^41^. It is unclear whether elevated IL-10 affect DME or it is elevated for compensatory immune modulation. However BCVA and CST are closely related and significant parameters of disease activity in DME^41^. Thus, more studies of IL-10 role in DME are required.

The highest IOP values were observed 2.15 ± 0.66 months after implantation, with an average increase of 4.96 ± 2.94 mmHg. In all, 13 patients (27.66%) had an IOP > 21 mmHg, so we prescribed anti-glaucoma agents and the IOP was subsequently well-controlled in all patients during the follow-up period. In these cases, the highest IOPs occurred between 1 month and 3 months after implantation. One previous study reported that an IOP-lowering medication was used by 41.5% of patients who received a dexamethasone intravitreal implant^16^. Another study reported that 88 of 377 patients showed ocular hypertension, defined as an IOP > 25 mmHg and/or an IOP increase >10 mmHg; furthermore, the IOP increase was associated with the implant position in the vitreous^42^. Because patients need to be treated for IOP increases, it is important to identify periods of higher IOP. According to our results, the first published data on this topic, clinicians should check the IOP more carefully between 1–3 months after implantation.

In this study we investigated the DME status using aqueous humor. Although analysis with vitreous samples could reflect retinal status more accurately, obtaining vitreous samples is very invasive or requires vitrectomy^43,44^. And aqueous humor is homogeneous while vitreous could not be depending on posterior vitreous detachment status. Additionally, many studies have previously proved that the aqueous humor could reflect retinal status; levels of many cytokines or growth factors are changed with retinal hypoxia or inflammation and after treatments^26,41,45–47^.

Our study had some limitations. First, we did not use OCT angiography or fluorescein angiography to evaluate macular status, including the ischemic status of patients in detail. Second, changes in the levels of aqueous biomarkers after dexamethasone treatment would have aided the evaluation of responses to these agents^48^, but we did not determine these parameters. Third, our sample size was relatively small. Although we tried additional analyses to find out factors associated with BCVA, but we could not get any significant result.

In summary, the duration of action of intravitreal dexamethasone implants in DME patients was associated with aqueous IL-8 levels and the number of HF using OCT.

## Methods

We followed all relevant tenets of the Declaration of Helsinki. This was a prospective study, and the protocol was approved by the institutional review/ethics board of the Catholic University of Korea (protocol number: VC16TISI0116). All participants gave written informed consent for the use of their clinical records.

### Study population

We enrolled naïve DME eyes with a CST > 300 µm from 2016 to 2018. Study participants were at least 18 years of age, had type II diabetes, and had received no anti-VEGF treatment or steroid treatments previously. The exclusion criteria included retinal degeneration, glaucoma, and macular edema attributable to other causes. We also excluded eyes with histories of prior ocular conditions, such as uveitis or intraocular surgery, including cataract surgery, which could influence enzyme levels in the aqueous humor.

### Study design

We measured glycated hemoglobin levels, and all patients underwent full ophthalmic examinations, including measurements of the BCVA, IOP, and a dilated fundus examination. Macular thickness was measured via OCT (Cirrus High-Definition OCT; Carl Zeiss Meditec, Dublin, CA, USA), and the axial length was measured using an IOL Master instrument (Carl Zeiss Meditec).

The HF, measured as the longest diameter of HF limited to a range of 20–50 μm, were manually measured within 1,500 µm, and ellipsoid zone (EZ) disruptions were manually measured within 1,000 µm using a horizontal scan centered on the fovea^36,49,50^. EZ disruptions were graded as 0 when intact, 1 in cases of focal disruption ≤200 µm in length, and 2 in cases of disruption >200 µm in length.

We placed a dexamethasone implant (Ozurdex^®^; Allergan, Irvine, CA, USA), and monitored all the patients with one month interval. We checked fundus, BCVA, CST, IOP, and any adverse events at every visit until DME recurrence with a CST >300 µm.

### Assays of cytokines and growth factors

We compared the levels of IL-1β, -8, -10, and -17; placental growth factor (PlGF); and VEGF in the aqueous humor. Concentrations of IL-1β, -8, -10, and -17; PlGF; and VEGF of the aqueous humor from the anterior chamber were measured using bead-immobilized antibodies. Aqueous humor samples were mixed with Calibrator Diluent RD6–52 and added to the bead preparations. A Luminex-x-MAP technique (Luminex, Austin, TX, USA) was used for reading. The detection limits and dynamic ranges are as follows: 0.8 pg/mL with a dynamic range to 3,950 pg/mL for IL-1β, 1.8 pg/mL with a dynamic range to 1,140 pg/mL for IL-8, 1.6 pg/mL with a dynamic range to 890 pg/mL for IL-10, 1.8 pg/mL with a dynamic range to 2,090 pg/mL for IL-17, 1.9 pg/mL with a dynamic range to 470 pg/mL for PlGF, and 2.1 pg/mL with a dynamic range to 2,170 pg/mL for VEGF. All values under the lower limit of detection were considered zero values.

### Statistical evaluation

All statistical analyses were performed using SPSS statistical software for Windows, version 21.0 (SPSS, Chicago, IL, USA). We used linear regression analyses to identify factors associated with the level of CST reduction and period from intravitreal dexamethasone implantation to the recurrence of DME. Additionally, we used logistic regression analyses to identify factors related to a shorter duration [<4 months (median value of duration of action in this study)] of action of dexamethasone implantation. The level of statistical significance was set at  p < 0.05.

## Data Availability

The datasets generated during and/or analysed during the current study are available from the corresponding author on reasonable request.
